# Cardioprotective effect of St. Thomas’ Hospital No. 2 solution against age-related changes in aquaporin-7-deficient mice

**DOI:** 10.1007/s11748-023-01975-y

**Published:** 2023-09-11

**Authors:** Masahiro Fujii, Ryuzo Bessho, Toshio Akimoto, Yosuke Ishii

**Affiliations:** 1 Cardiovascular Surgery, Nippon Medical School Chiba Hokusoh Hospital, 1715 Kamagari, Inzai, Chiba 270-1694 Japan; 2 Laboratory Animal Science, Nippon Medical School, 1-1-5 Sendagi, Bunkyo, Tokyo 113-8603 Japan; 3 Cardiovascular Surgery, Nippon Medical School, 1-1-5 Sendagi, Bunkyo, Tokyo 113-8603 Japan

**Keywords:** Mouse, Aquaporin-7, Cardioplegia, Ischemia–reperfusion

## Abstract

**Objective:**

This study aimed to investigate whether St. Thomas’ Hospital No. 2 solution (STH2) is equally effective in both young and aged aquaporin-7-knockout (AQP7-KO) mice and the mechanisms by which the intra-myocardial adenosine triphosphate (ATP) content is altered during ischemia without aquaporin-7.

**Methods:**

In study 1, isolated hearts of male wild-type (WT) and AQP7-KO mice (< 12 weeks old) were Langendorff perfused with 5-min STH2 prior to a 20-min global ischemia (GI) or 25-min GI without STH2. Similarly, in Study 2, hearts from WT and AQP7-KO mice (≥ 24 weeks old) were subjected to 2-min STH2 infusion prior to GI. In study 3, intra-myocardial ATP content was compared before (sham) and after (control or STH2) ischemia in mature WT and AQP7-KO mice.

**Results:**

In study 1, troponin T levels (ng/g wet weight) of WT and AQP7-KO hearts were significantly lower in the STH2 groups (75.6 ± 45.9 and 80.2 ± 52.2, respectively) than in the GI groups (934.0 ± 341.1 and 1089.3 ± 182.5, respectively). In Study 2, troponin T levels in aged WT and AQP7-KO mice were 566.5 ± 550.0 and 547.8 ± 594.3, respectively (*p* = 0.9561). In Study 3, ATP levels (μmol/g protein) in the sham, control, and STH2 AQP7-KO mice groups were 4.45, 2.57, and 3.37, respectively(*p* = 0.0005).

**Conclusions:**

The present study revealed the cardio-protective efficacy of STH2 in an experimental model of isolated AQP7-KO young and aged murine hearts. Further, STH2 preserved intra-myocardial ATP during ischemia with Krebs–Henseleit buffer perfusion in the Langendorff setting.

## Introduction

Aquaporins (AQPs) are transmembrane proteins that regulate transcellular water flow. Mammalian AQPs are present in many tissues and organs, such as the kidneys, brain, lungs, digestive system, eyes, and skin [1]. AQPs are involved in diverse physiological and pathophysiological processes, particularly in cerebral ischemia, congestive heart failure, hypertension, and angiogenesis. Nevertheless, many effects of cardiac AQPs are yet to be elucidated [1]. To date, 13 AQPs have been identified in mammals (AQP0–12, encoded by the genes AQP0–AQP12): they are divided into three groups: the classical group (AQP1, 2, 4, and 5) that selectively transfers water; the aqua-glycero-porin group (AQP3, 7, 9, and 10) that transfers both water and small molecules (e.g., glycerol); and the unorthodox group that includes un-categorizable AQP6, 8, 11, and 12 [2]. Several AQP subtypes have been discovered in the cardiac tissues of various animals and humans using real-time polymerase chain reaction [3–5].

Cardiomyocytes require fatty acids and glucose for energy production [6, 7]. A recent study showed that glycerol was an important cardiac energy substrate and that AQP7 was a glycerol facilitator [8]. AQP7 deficiency increases myocardial infarction size and apoptosis after ischemia [9]. AQP7 expression is downregulated in obese patients [6]. Analysis of the AQP7 sequence in 160 human subjects revealed three missense mutations, one of which was the AQP7-G264V protein with impaired glycerol and water transport functions [10]. We previously reported the relationship between AQP7 and myocardial injury against normothermic global ischemia (GI) and elucidated the efficacy of St. Thomas’ Hospital No. 2 solution (STH2), a hyperkalemic cardioplegia, in a mature murine heart with AQP7 deficiency [11]. Unfortunately, AQP7-KO mice exhibited obesity and insulin resistance after 12 weeks [12]. Further investigations comparing the effects of STH2 on ischemia–reperfusion injury among different age groups are needed. To better understand these associations, the present study aimed to investigate whether STH2 was equally effective in young and aged AQP7-KO mice and to elucidate the mechanisms by which intra-myocardial adenosine triphosphate (ATP) content was altered during ischemia in AQP7-KO mice.

## Methods

### Ethics statements

This study was approved by the Animal Ethics Committee of the Nippon Medical School (no.: 2020-103, 2021-17). All animals received humane care in compliance with the “Principles of Laboratory Animal Care” formulated by the National Society for Medical Research and the “Guide for the Care and Use of Laboratory Animals” published by the National Institute of Health (NIH) (NIH publication number: 85-23, revised 1996).

### Animals

AQP7-deficient mice (B6;129-Aqp7 < tm1Tfun >) were generated and maintained as previously described [13]. Frozen sperm provided by the RIKEN BioResource Research Center (Ibaragi, Japan) was restored and used for breeding at the Jackson Laboratory in Japan. Six-week-old wild-type (WT) and AQP7-KO mice (C57BL/6N) were brought to our laboratory, fed regular chow, and maintained in 22 °C rooms with a 12/12-h dark/light cycle (light cycle: 8 AM–8 PM) until use. The genotypes of *Aqp7*-KO mice were determined through PCR of the genomic DNA extracted from their ears. Genotyping primers for the Aqp7 WT allele were as follows: forward 5′-CTTGGTCTGCTGCTTCAGGTC-3′ and reverse 5′-CAGAGTCCCTCTCACGTCAC-3’. The expected size of the WT band was 1288 bp. Genotyping primers for the *Aqp7* null allele were as follows: forward 5′-CTTGGTCTGCTGCTTCAGGTC-3′ and reverse 5′-GCAATCCATCTTGTTCAATGGCC-3′. The expected size of the KO band was 800 bp. All mice used in this study were generated by intercrossing Aqp7 heterozygous (+ / −) mice. Male and female Aqp7-KO mice and their WT littermates were used as controls.

### Heart isolation and perfusion

The mice were anesthetized by intraperitoneal injection of sodium pentobarbital (100 mg/kg) mixed at a ratio of 50:50 with an anticoagulant (heparin, 1000 IU/kg). The hearts were excised and immersed in cold (4.0 °C) Krebs–Henseleit bicarbonate buffer (KHB). The aorta was cannulated rapidly and perfused with KHB in Langendorff mode at a constant pressure of 80 mmHg and continuously gassed with a mixture of 95% oxygen and 5% carbon dioxide to obtain a pH of 7.4 at 37 °C.

After removing the left atrial appendage, a fluid-filled balloon catheter attached to a pressure transducer was introduced into the left ventricle via the mitral valve. The balloon was inserted until the left ventricular end-diastolic pressure (LVEDP) was between 4 and 8 mmHg, and the balloon volume was not altered thereafter. All pressure transducers were connected to a PowerLab system (AD Instruments, Dunedin, New Zealand), which continuously recorded throughout the experiment. Each heart was placed in a thermostatically controlled water-jacketed chamber to maintain its temperature at 37.0 ± 0.2 °C.

All hearts were equilibrated for 20 min of aerobic perfusion, and baseline readings of left ventricular systolic pressure (LVSP, mmHg), LVEDP (mmHg), heart rate (beats/min), and coronary flow (mL/min) were measured. Left ventricular developed pressure (LVDP) was calculated as the difference between the LVSP and LVEDP. At the time of the baseline readings, hearts were excluded if the acceptable ranges of LVDP > 50 mmHg, heart rate > 300 beats/min, and coronary flow < 1 mL/min, > 5 mL/min were not met.

### Perfusion medium and drugs

The KHB consisted of 118.5 mmol/L NaCl, 25.0 mmol/L NaHCO_3_, 4.8 mmol/L KCl, 1.2 mmol/L MgSO_4_, 1.18 mmol/L KH_2_PO_4_, 1.4 mmol/L CaCl_2_, and 11.0 mmol/L glucose. The KHB was prepared daily and filtered through a 5-µm cellulose nitrate filter before use. STH2 (Miotector®; FUSO Pharmaceutical Industries, Ltd., Osaka, Japan) was prepared daily with the following composition (in mmol/L): 110.0 NaCl, 16.0 MgCl_2_·2H_2_O, 16.0 KCl, 1.2 CaCl_2_·2H_2_O, and 10.0 NaHCO_3_. The pH was adjusted to 7.8 at 37 °C, and the solution was filtered through a 5-µm cellulose nitrate filter before use.

### Perfusion protocol

**Fig. 1:**
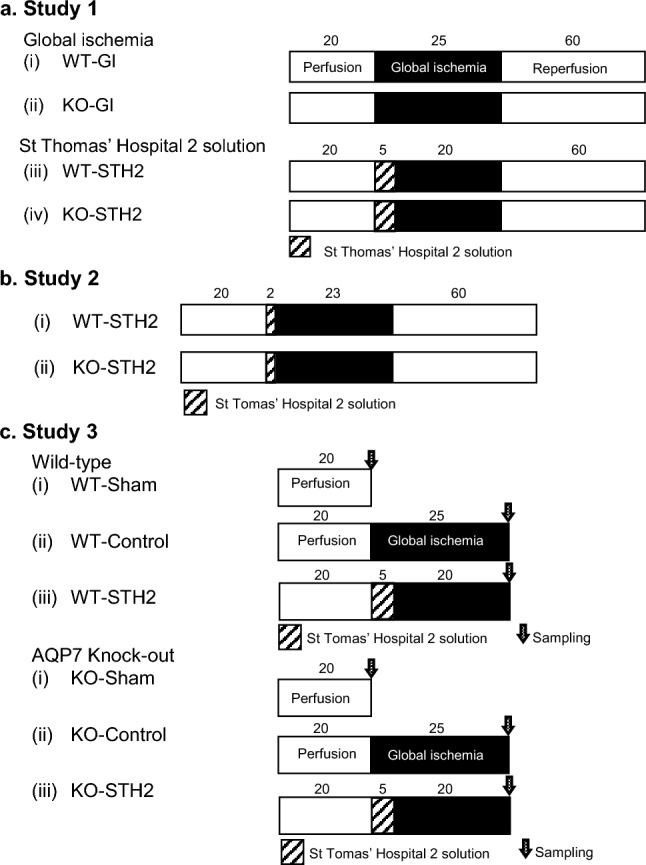
Experimental perfusion protocol. In this protocol, the hearts are aerobically Langendorff perfused at a constant pressure equivalent to 80 mmHg during the experiment using 20 min of stabilization before global ischemia. **a** Study 1. Each heart in both WT and KO young mice is used. Protocol abbreviations: (i) WT-GI, no treatment with 25 min of global ischemia, followed by 60 min of reperfusion; (ii) KO-GI, no treatment with 25 min of global ischemia, followed by 60 min of reperfusion, (iii) WT-STH2, 5-min pre-treatment with STH2 infusion and 20 min of global ischemia, followed by 60 min of reperfusion; and (iv)KO-STH2, 5-min pre-treatment of STH2 infusion and 20 min of global ischemia, followed by 60 min of reperfusion. WT, wild-type; KO, aquaporin-7 knockout; STH2, St. Thomas’ Hospital 2 solution. **b** Study 2. The hearts are pretreated with 2-min STH2 before 23 min of global ischemia. Protocol abbreviations: (i) WT, wild-type mice; (ii) KO, aquaporin-7 knockout mice. **c** Study 3. The hearts were categorized in to 3 groups. Protocol abbreviations: (i) Sham, 20-min perfusion only; (ii) Control, no treatment and 25 min of global ischemia; (iii) STH2, 5-min pre-treatment with STH2 infusion and 20 min of global ischemia. STH2, St. Thomas’ Hospital No. 2 solution

The experimental protocol was conducted in three stages, including two studies to investigate the age-dependent effects of STH2 in young or aged AQP7-KO mice and another study to assess the dynamics of intra-myocardial ATP against ischemia in AQP7-deficient mature mice (Fig. 1).

#### Study 1: to investigate whether STH2 influenced the recovery of cardiac function in young mice

This study used 9–12-week-old young animals. For all perfusion protocols, each heart in both WT and AQP7-KO young mice was subjected to a 20-min equilibration period of aerobic KHB perfusion at 37 °C. After equilibration, hearts were randomly assigned to one of two groups (*n* = 6 per group): 25 min of GI (WT-GI, KO-GI) or pre-treatment with 5-min of STH2 infusion and 20-min of GI (WT-STH2, KO-STH2), followed by 60-min of reperfusion. The final recovery of myocardial function and coronary flow was measured and compared within each group. To evaluate the effects of STH2 on myocardial injury, coronary effluents were collected, and troponin T levels [expressed as ng/g heart wet weight (ng/g wt)] were measured using an electro-chemiluminescence immunoassay [11]. Myocardial edema was measured based on the water content of each heart in a microwave oven to dry the water, as previously reported [11].

#### Study 2: to investigate whether STH2 influenced the recovery of cardiac function in aged mice

This study used 24–27-week-old aged animals. After a 20-min equilibration period in both WT and AQP7-KO aged murine hearts, the hearts were pretreated with 2-min STH2 infusion and then subjected to 23-min GI followed by 60-min reperfusion. The final recovery of myocardial function and coronary flow was measured and compared between the groups. To evaluate the effects of STH2 on myocardial injury, coronary effluents were collected, and troponin T levels [expressed as ng/g heart wet weight (ng/g wt)] were measured using an electro-chemiluminescence immunoassay.

#### Study 3: to measure the changes of intra-myocardial ATP against ischemia in AQP7-deficient mature mice

This study used 15–21-week-old mature adult animals. For all perfusion protocols, the hearts in WT and AQP7-KO matured mice were randomly assigned to one of three groups (*n* = 3 per group): a 20-min period of aerobic KHB perfusion only at 37 °C (Sham), a 25-min period of GI after 20-min equilibration, or a pre-treatment with 5 min of STH2 infusion after 20-min equilibration and 20-min GI. ATP was extracted from the homogenized myocardium using an AMERIC-ATP kit (Applied Medical Enzyme Research Institute Corporation, Tokushima, Japan) and measured using a luminometer (Lumat LB9510, Berthold Japan K.K., Sumida-ku, Tokyo, Japan). We compared the amount of ATP per unit of protein in the cardiac tissue before and after ischemia with and without STH2.

### Statistical analysis

Post-ischemic recovery of LVDP, heart rate, and coronary flow was expressed as a percentage of the baseline values; LVEDP (mmHg) and troponin T levels (ng/g wt) were expressed as absolute values. All data were expressed as the mean ± standard deviation (SD). Continuous variables were compared between two groups using Student’s *t*-test or the Mann–Whitney *U* test as appropriate. Categorical variables were compared between two groups using χ^2^ test or Fisher exact test as appropriate. Comparisons between the groups were assessed for significance by analysis of variance (ANOVA) or two-way repeated-measures ANOVA as appropriate. If significance was established, and post hoc analysis was performed by means of the Tukey test, which allowed for multiple comparisons. All statistical analyses were performed using JMP, version 10.0 (SAS Inc., Cary, NC, USA). All statistical tests were two tailed, with *p* < 0.05 regarded as statistically significant.

## Results

### Influence of STH2 on the recovery of cardiac function in young mice

**Fig. 2:**
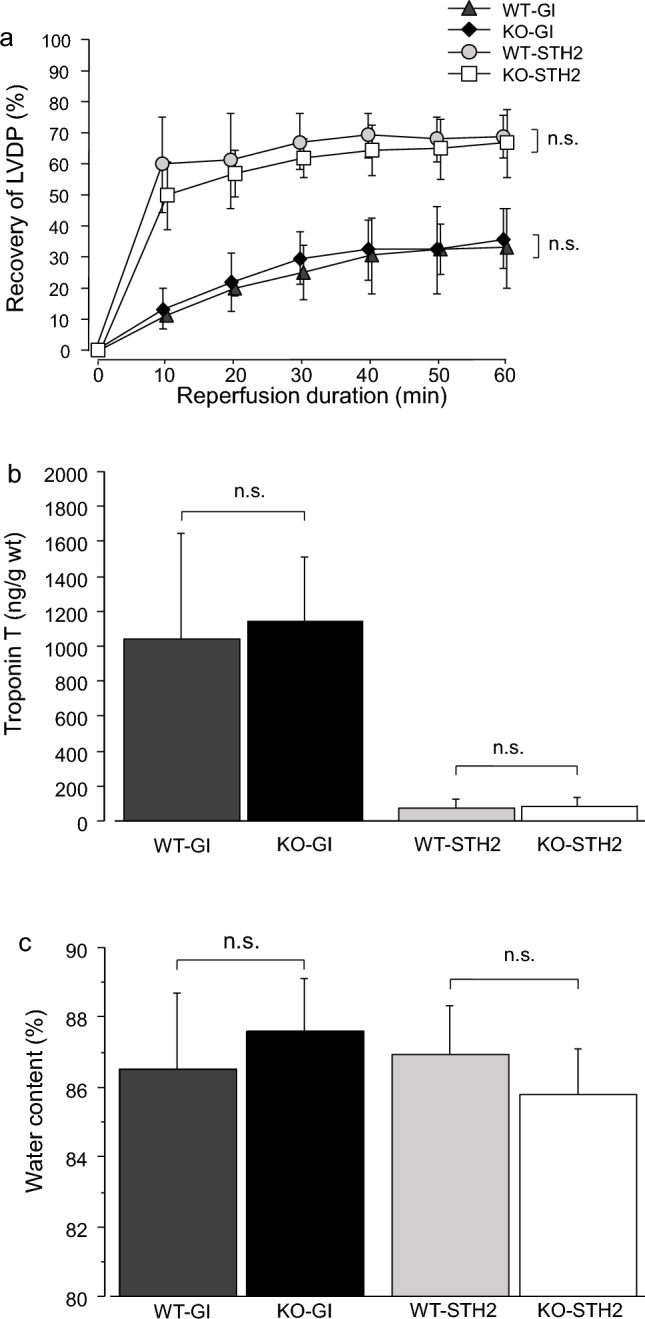
**a** Recovery of left ventricular developed pressure (LVDP) according to the reperfusion duration (min) in Study 1, expressed as a percentage of the baseline value. Values are shown as means ± SDs (*n* = 6 hearts/group). Dark gray triangles, wild-type (WT) mice in the control group; light gray circles, WT mice in the St. Thomas’ Hospital No. 2 solution (STH2) group; black rhombus, aquaporin-7 knockout (AQP7-KO) mice in the control group; white squares, AQP7-KO mice in the STH2 group. **b** Total troponin T leakage (expressed as ng/g wet weight) during 60 min of reperfusion in Study 1. Values are the mean ± SD (*n* = 6 hearts/group). **c** Water content (%). Values are shown as means ± SDs (*n* = 6 hearts/group)

There were no significant differences in any of the parameters within each group. The baseline and the final recoveries of all parameters are shown in Table 1. The post-ischemic recovery of LVDP in both GI groups was low (reaching a plateau value of approximately 30% of the baseline), whereas it was highly and rapidly recovered in the rats that received STH2 before GI, reaching approximately 65% (Fig. 2A). The troponin T levels (ng/g wet weight) in the WT and AQP7-KO groups without STH2 were 934.0 ± 341.1 and 1089.3 ± 182.5, respectively. Meanwhile, the troponin T levels in the WT and KO groups with STH2 were 75.6 ± 45.9 and 80.2 ± 52.2, respectively (Fig. 2B). There was no significant difference in water content within each group (Fig. 2C).

**Table 1:** Baseline values of LVDP, heart rate, coronary flow, and LVEDP in Study 1

Group		GI			STH2		
		WT	KO	*p* value	WT	KO	*p* value
HR	Baseline (/min)	385.8 ± 34.5	406.7 ± 49.3	0.4162	420.0 ± 41.4	385.0 ± 42.4	0.1789
	Recovery (%)	94.0 ± 10.5	91.2 ± 8.5	0.6277	90.8 ± 5.0	97.0 ± 17.3	0.4212
LVDP	Baseline (mmHg)	72.2 ± 21.7	70.4 ± 12.4	0.8674	66.7 ± 11.1	78.1 ± 17.5	0.2104
	Recovery (%)	32.9 ± 12.6	35.9 ± 9.8	0.6579	66.7 ± 11.1	68.8 ± 6.9	0.7071
LVEDP	Baseline (mmHg)	5.1 ± 0.7	5.8 ± 1.1	0.2801	5.0 ± 0.5	4.8 ± 1.5	0.7399
	Final (mmHg)	65.1 ± 10.9	62.9 ± 12.4	0.7584	17.9 ± 9.7	26.4 ± 13.9	0.2476
CF	Baseline (mL/min)	1.2 ± 0.8	1.3 ± 1.3	0.9145	1.3 ± 0.2	1.2 ± 0.5	0.5424
	Recovery (%)	107.9 ± 38.7	107.2 ± 27.1	0.9738	82.2 ± 18.4	89.5 ± 23.6	0.5652

Data are presented as the mean ± standard deviation

*GI* global ischemia, *STH2* St. Thomas’ Hospital solution 2, *WT* wild-type mice, *KO* aquaporin-7 knockout mice, *HR* heart rate, *LVDP* left ventricular developed pressure, *LVEDP* left ventricular end-diastolic pressure, *CF* coronary flow

### STH2 influence on the recovery of cardiac function in aged mice

**Fig. 3:**
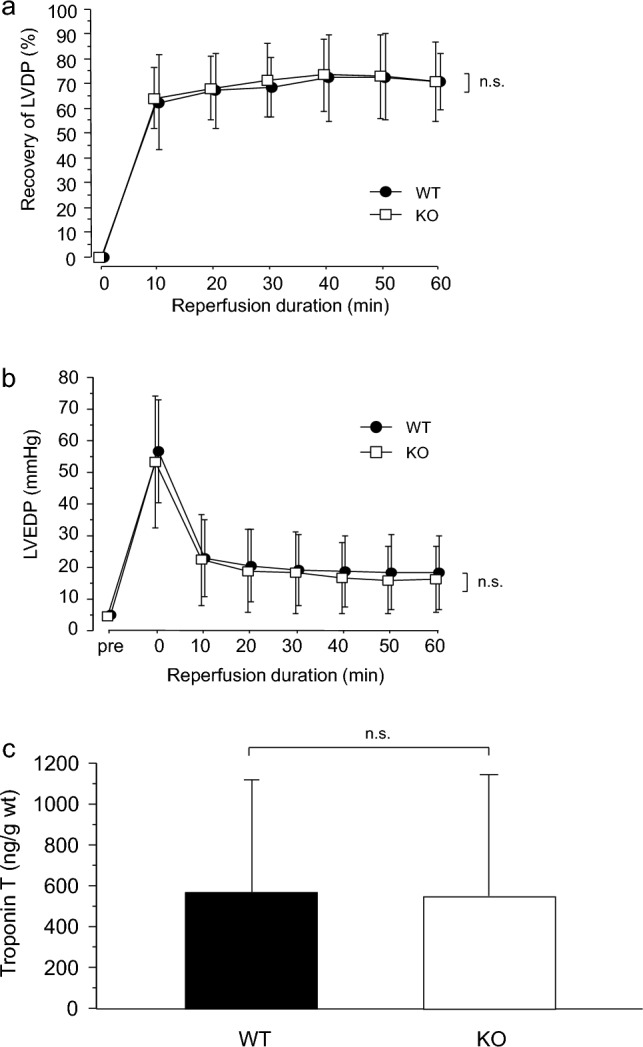
**a** Recovery of left ventricular developed pressure (LVDP) according to the reperfusion duration (min) in Study 2, expressed as a percentage of the baseline value. Values are shown as mean ± SD (*n* = 6 hearts/group). Filled circles, wild-type (WT) mice; open squares, AQP7-knockout (KO) mice. **b** Changes in LVEDP values during reperfusion. Values are shown as mean ± SD (*n* = 6 hearts/group). Filled circles, WT mice; open squares, AQP7-KO mice. **c** Total troponin T levels (expressed as ng/g wet weight) during 60 min of reperfusion in Study 2. Values are shown as means ± SDs (*n* = 6 hearts/group)

The baseline values and the percentages of recovery of the heart rate, LVDP, coronary flow, and final LVEDP are presented in Table 2. There were no significant differences between the WT and AQP7-KO aged groups. The hearts in the STH2 groups recovered rapidly, reaching a significantly higher plateau of approximately 70% after 30 min of reperfusion (Fig. 3A). The recovery of the LVEDP was similar to that of the LVDP (Fig. 3B). The troponin T levels (ng/g wet weight) in the AQP7-KO and WT groups were similar (Fig. 3C).

**Table 2:** Baseline values and percentage of recovery of LVDP, heart rate, coronary flow, and final LVEDP value in Study 2

		WT	KO	*p* value
HR	Baseline (/min)	324.7 ± 25.5	336.8 ± 43.5	0.5617
	Recovery (%)	98.1 ± 7.0	106.6 ± 14.1	0.2176
LVDP	Baseline (mmHg)	84.4 ± 13.8	70.8 ± 10.4	0.0820
	Recovery (%)	70.7 ± 11.6	70.8 ± 16.2	0.9889
LVEDP	Baseline (mmHg)	4.9 ± 0.7	4.6 ± 0.7	0.4051
	Final (mmHg)	18.5 ± 11.7	16.3 ± 10.4	0.7386
CF	Baseline (mL/min)	5.0 ± 3.0	3.4 ± 2.0	0.2809
	Recovery (%)	109.8 ± 22.5	99.8 ± 25.4	0.4876

Data are presented as the mean ± standard deviation

*WT* wild-type mice, *KO* aquaporin-7 knockout mice, *HR* heart rate, *LVDP* left ventricular developed pressure, *LVEDP* left ventricular end-diastolic pressure, *CF* coronary flow

### Changes of intra-myocardial ATP against ischemia in AQP7-deficient matured mice

**Fig. 4:**
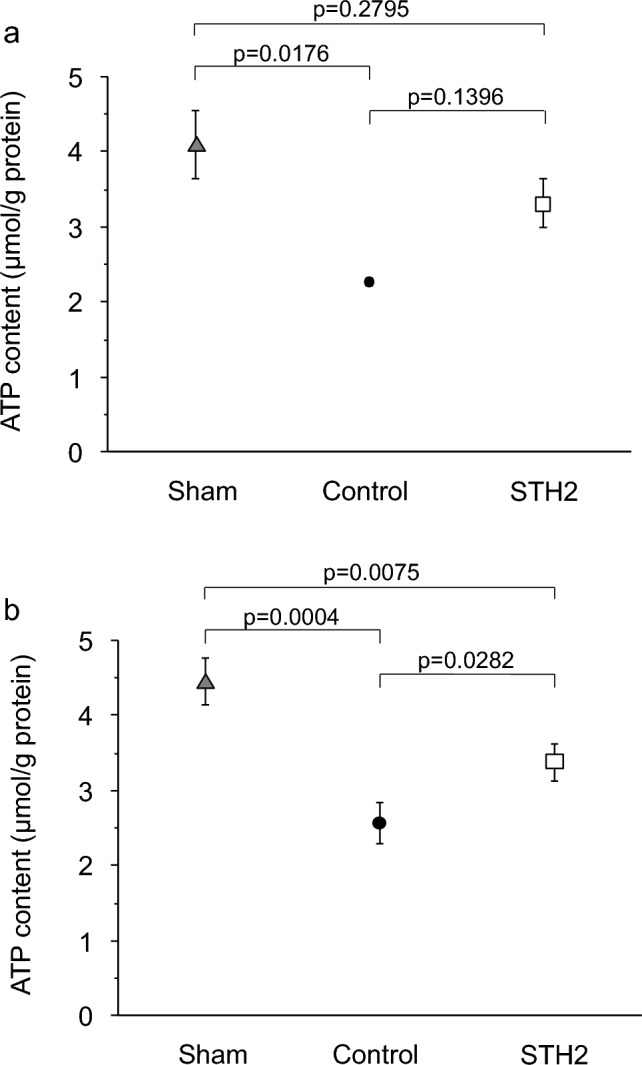
**a** Levels of intra-myocardial adenosine triphosphate (ATP) in wild-type mice in Study 3. Values are shown as means ± SDs (*n* = 3 hearts/group). Sham, 20-min perfusion only; Control, global ischemia after 20-min equilibration; STH2, 5-min pre-treatment with St. Thomas’ Hospital solution No. 2 infusion before 20-min of global ischemia. **b** Levels of intra-myocardial ATP of AQP7-knockout mice in Study 3. Values are shown as means ± SDs (*n* = 3 hearts/group). Sham, 20-min perfusion only; Control, global ischemia after 20-min equilibration; STH2, 5-min pre-treatment with St. Thomas’ Hospital solution No. 2 infusion before 20-min of global ischemia

The amounts of intra-myocardial ATP (μmol/g protein) after GI in the control group were significantly lower than those before GI in the sham group in both the WT and AQP7-KO mice. This reduction was ameliorated by STH2 treatment (Fig. 4).

## Discussion

The present study investigated the relationship between AQP7 and myocardial injury against normothermic GI and elucidated the efficacy of hyperkalemic cardioplegia in young and aged murine hearts with AQP7 deficiency. We found that changes in intra-myocardial ATP content in AQP7-KO mice were comparable to those in WT mice in the Langendorff perfused system. These findings indicate that AQP7 deficiency does not influence the myocardial protective effect of STH2 in hyperkalemia depolarized cardioplegia.

AQP7 is a membrane protein that is a member of the glycerol and water molecule-transporting aqua-glycero-porin family. Accordingly, AQP7 is also involved in the passive transfer of other small uncharged solutes, such as urea, purines, arsenite, ammonia, and hydrogen peroxide [14]. AQP7 deficiency has also been shown to result in insulin resistance [15]. Some AQPs have been recently found to function as ion channels, and the effects of divalent cations on the ionic conductance of AQPs have been studied. Kourghi et al. reported that human AQP1-expressing oocytes showed little effect of Mg^2+^, in contrast to moderate inhibition by Ca^2+^ [16]. Given that STH2 contains magnesium chloride, which inhibits Ca^2+^ channels and provides additional cardio-protective effects, it is an important clinical question to understand whether Mg^2+^ affects the regulatory function of AQP7.

AQP7 is highly expressed in white and adipose tissue. Meanwhile, AQP7 is weakly expressed in the heart, skeletal muscle, and kidneys. Glycerol produced by the hydrolysis of triglycerides in adipocytes is efficiently released by AQP7 [17]. Méndez-Giménez et al. [18] found increased AQP7 expression in the adipose tissues of experimental rats with diet-induced obesity undergoing bariatric surgery. Another mouse analysis showed that AQP7 mRNA levels in the quadriceps femoris muscles were significantly higher in mice with diet-induced obesity than in control chow-fed mice [19]. Immuno-histochemical analysis of the muscles of mice with diet-induced obesity showed enhanced expression of AQP7 at the myofiber surface membranes, suggesting that AQP7 facilitated the secretion of glycerol from myocytes. A similar phenomenon was observed in another study using obese leptin-deficient ob/ob mice [20].

Wakayama et al. [21] demonstrated that AQP7 mRNA expression in the myocardium, as well as in the skeletal muscle, was two times higher in db/db mice than in WT mice. Interestingly, Hibuse et al. [12] found no difference in body weight between young WT and AQP7-KO, although AQP7-KO mice developed obesity and insulin resistance at age 12 weeks due to increased glycerol kinase (Gyk) activity [12]. In the present study, no remarkable difference was found between the body weight of young WT and AQP7-KO mice (28.8 ± 1.9 g vs 27.7 ± 2.2 g). Meanwhile, in aged mice, although the average body weight was still not significantly different (39.2 ± 6.7 g in WT mice vs 42.5 ± 2.1 g in AQP7-KO mice), there was smaller inter-individual variability in KO mice than in WT mice. A recent clinical study on subcutaneous abdominal fat biopsies reported that AQP7 expression in adipose tissue was lower in obese subjects than in lean subjects [22]. The gene expression of AQP7 in different animal species and organs during obesity should be further investigated.

Hibuse et al. [8] reported that myocardial glycerol content under normal feeding conditions was significantly lower in AQP7-KO mice than in WT mice. Furthermore, the cardiac ATP content was significantly lower in AQP7-KO mice than in WT mice [8]. They assessed energy metabolism to examine the consumption of energy substrates using isolated murine hearts perfused in the Langendorff mode and found no differences in palmitic acid or glucose consumption, whereas the glycerol consumption rate was significantly reduced in AQP7-KO mice. The intracellular ATP content increased with the addition of glucose, even under AQP7 knockdown. However, the increase in intracellular ATP content induced by the addition of glycerol was significantly blocked in AQP7-KO mice. The authors found that glycerol acted as an energy substrate in the heart via AQP7.

Ischemic conditions activate glycerol-3-phosphate dehydrogenase 2 (GPD2), which converts glycerol-3 phosphate to dihydroxyacetone phosphate to facilitate ATP synthesis from glycerol. The increase in glycerol-mediated ATP production under hypoxic conditions is suppressed by a GPD2 inhibitor in a dose-dependent manner [9]. In addition, Ishihama et al. [9] reported that AQP7 deficiency in mice increased myocardial infarct size after left coronary artery ligation and myocardial infarct-induced cardiac apoptosis in response to ischemia. Moreover, atrial natriuretic peptide levels in the heart, which increase upon cardiac dysfunction, were elevated 7 days after ligation in AQP7-KO mice [9]. In the present study, hearts were rapidly arrested with STH2, and the intracellular ATP content was significantly more preserved than in untreated hearts. As we used KHB-containing glucose rather than glycerol, further investigation is required using KHB with glycerol.

## Study limitations

The hearts were perfused with standard KHB, including glucose, without glycerol or palmitic acid, and thus, further studies are warranted to assess metabolic adaptation and nutrition-related conditions (i.e., a specific diet or starvation). Myocardial ischemic disease is a multifactorial process involving a spectrum of injuries that affect myocardial protection. However, the hearts used in this experimental study were obtained from healthy WT and AQP7-KO mice under normal feeding conditions. It is likely that any protective effect of STH2 cardioplegia might be different in hearts with ischemic injury, hypertrophy, or in older hearts. In addition, damaged hearts are likely to require prolonged periods of ischemia to correct a lesion; however, the ischemic duration adopted in this experimental study is relatively short. Hence, it is necessary to investigate the use of longer ischemic durations and repeated administration of cardioplegia in accordance with clinical conditions. Ischemic tolerance and AQP function may be altered under hypothermic conditions in normal clinical practice.

## Conclusion

The present study reveals the cardio-protective efficacy of STH2 in an experimental model of isolated AQP7-KO young and aged (> 6 months) murine hearts and the preservation of intra-myocardial ATP during ischemia by STH2. The findings suggest the safety of this cardiac-protective strategy even when the AQP7 gene is downregulated due to obesity or missense mutations.

## Data Availability

Data available on request.
